# A prototype pond water management system (dissolved oxygen, pH and temperature) for giant freshwater prawn farming in Pak Phanang, Southern Thailand

**DOI:** 10.1016/j.heliyon.2024.e31231

**Published:** 2024-05-14

**Authors:** Panyapong Songpayome, Supapron Sutin, Warawut Sukmak, Uraiwun Wanthong, Nunticha Limchoowong, Phitchan Sricharoen, Panjit Musik

**Affiliations:** a Program in Creative Innovation in Science and Technology, Faculty of Science and Technology, Nakhon Si Thammarat Rajabhat University, Nakhon Si Thammarat, 80280, Thailand; b Faculty of Veterinary Science, Rajamangala University of Technology Srivijaya, Nakhon Si Thammarat Campus, Nakhon Si Thammarat, 80240, Thailand; c Faculty of Science and Technology, Nakhon Si Thammarat Rajabhat University, Nakhon Si Thammarat, 80280, Thailand; d Department of Chemistry, Faculty of Science, Srinakharinwirot University, Bangkok, 10110, Thailand; e Division of Health, Cosmetic and Anti-Aging Technology, Faculty of Science and Technology, Rajamangala University of Technology Phra Nakhon, Bangkok, 10800, Thailand; f Department of Chemistry, Faculty of Medicine, Bangkokthonburi University, Bangkok, 10170, Thailand; g Center of Excellence for Ecoinformatics, School of Science, Walailak University, Nakhon Si Thammarat, 80160, Thailand

**Keywords:** Giant freshwater prawns, Smart water management system, IoT

## Abstract

Significant income was promised by giant freshwater prawn farming, which served as a key occupation for farmers. However, challenges were faced by traditional methods, including difficulties in selling prawns of incorrect sizes, limited market demand, low prices, and the risk of waterborne diseases. In pond-raised environments, these prawns were prone to diseases due to deteriorating environmental conditions, particularly poor pond bottoms, resulting in unsuitable water quality and vulnerability in prawn growth. Consequently, the application of water management technology in the prawn ponds becomes crucial to address these issues. This research aims to develop a prototype for a smart water management system, designed to regulate the water quality that significantly impacts the growth of Pak Phanang giant freshwater prawns within a pond. The equipment and tools utilized comprise ponds, water treatment systems, and smart water quality control systems. The investigation resulted in favorable findings, obtaining a total of 270 river prawns, each measuring an average length of 10–12 cm and weighing between 24 and 30 g. After a rearing period of 120 days in this system, 252 river prawns were successfully harvested, exhibiting a survival rate of 93.3 %. These prawns measured 20–25 cm in length and weighed between 190 and 268 g each, while remaining free from diseases. The results demonstrate that the developed system effectively manages water quality throughout the culture process, offering a valuable model to address the challenges faced by giant freshwater prawn farmers. The prawns obtained are of marketable size, and disease-free, enhancing the farmers' credibility with long-term benefits in the consumer market, in addition to their income generation potential.

## 1 Introduction

River prawns, scientifically known as giant freshwater prawns or *Macrobrachium rosenbergii*, are native to tropical rivers and estuaries across the Indo-Pacific region. These prawns hold significant commercial value worldwide [1,2]. Due to their popularity among consumers, river prawns living in natural water sources serve as crucial economic assets for local fishermen and communities. Typically, local fishermen capture river prawns with sizes ranging from 20 to 40 individuals per kilogram. However, consumers prefer larger prawns, despite the higher unit price. The most sought-after size falls within the range of 5–7 prawns per kilogram. In the Pak Phanang Basin of Nakhon Si Thammarat Province in Southern Thailand, local fishermen can sell river prawns at prices as high as 110–140 baht per kilogram for sizes of 35–40 prawns per kilogram, while sizes of 5–7 prawns per kilogram can fetch prices of 500–600 baht per kilogram [3]. Consequently, local fishermen aim to enhance the size of river prawns by rearing them in ponds after capturing them in natural water sources. However, most prawn farmers rely on traditional methods to promote prawn growth, resulting in inefficiencies in terms of prawn quantity (survival rate) and quality (growth to commercial size). To ensure the investment is worthwhile and yields high returns, farming systems can be modified and enhanced with technology to improve efficiency [3,4].

The integration of technology to enhance the efficiency of river prawn farming necessitates an understanding of the factors influencing prawn growth. Previous studies have highlighted the significance of dissolved oxygen (DO), pH, and temperature in prawn farming [[5], [6], [7], [8]]. Giant freshwater prawn farmers can benefit from utilizing sensor technology to monitor environmental parameters such as DO, pH, and temperature. The sensor system is connected to a microcontroller and an IoT system. In the event of any deviations in these parameters, the system will promptly notify the farmer [9,10].

Giant freshwater prawns raised in ponds, often suffer from diseases due to the deteriorating environmental conditions. The unsuitable bottom condition and water quality hinder their growth and weakens their immunity, making them susceptible to bacterial infections [7]. As natural water-loving creatures, river prawns require high levels of dissolved oxygen and a constant flow of clear water. Therefore, it is crucial to find methods that simulate natural environments to rear prawns and minimize the occurrence of diseases. Traditional clay ponds (referring to ponds constructed using locally sourced natural clay) utilized for cultivating river prawns demand thorough preparation, such as pond drying, regulating the pond's pH levels, and managing oxygen levels to ensure adequate oxygen supply for prawn viability [11,12].

In the present day, the Internet of Things (IoT) applications are increasingly employed in agriculture to assist farmers in recording agricultural data and controlling agricultural processes [[13], [14], [15], [16], [17]]. The use of IoT for smart water management in prawn farms offers a new solution for fishermen to monitor and control water quality data, providing convenience and saving time [4,10]. This research aims to develop a prototype of a smart water management system to regulate water quality, which significantly influences the growth of Pakpanang giant freshwater prawns in Southern Thailand. The prototype comprises a water quality monitoring system, a water treatment and control system, and an IoT system. The objective is to rear wild-caught freshwater prawns in the prototype pond, achieving a weight of 200–300 g per individual, while studying the growth and survival rate of the river prawns in this controlled environment.

## 2 Materials and methods

### 2.1 Design and construction of management systems

**Fig. 1:**
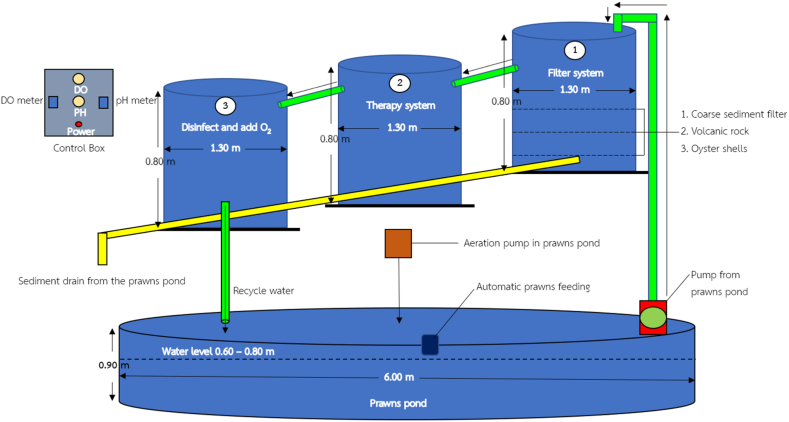
The prototype of smart water management system.

This work revolved around the design and application of the three-tier filter system, which was linked via water quality sensors to manage the stability of the aquaculture pond, along with the above-ground artificial pond, aimed at supporting the health and growth of productive giant freshwater prawns. From Fig. 1, the prawn ponds were constructed using a water proof membrane of cylindrical canvas made of polyethylene material, with a wall height of 0.9 m and a diameter of 6 m. The rigid structural-support frame of the ponds was constructed with PVC material and placed within a roofed enclosure, allowing for 70 % sunlight filtration while ensuring adequate ventilation from all directions. The ponds were filled with water, reaching a depth of 70 cm.

**Fig. 2:**
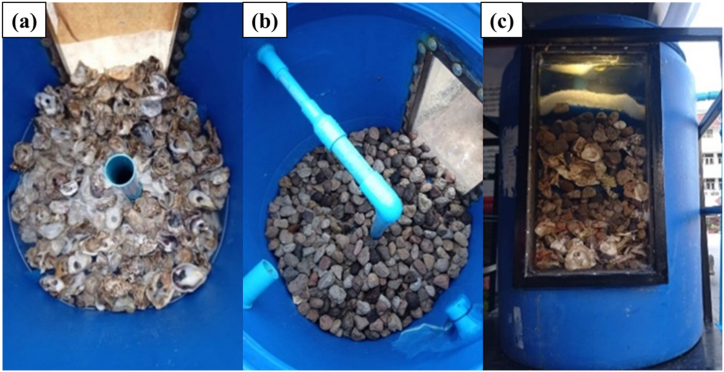
The water treatment system of tank 1 (filter system). (a) 1st layer (bottom), (b) 2nd layer and (c) side view of 1st tank.

**Fig. 3:**
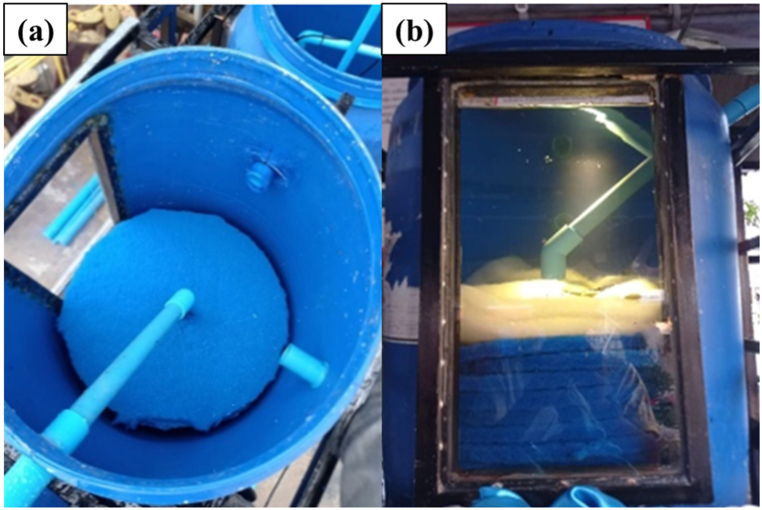
The water treatment system of 2nd tank (therapy system). (a) 1st layer (bottom), and (b) side view of 2nd tank.

**Fig. 4:**
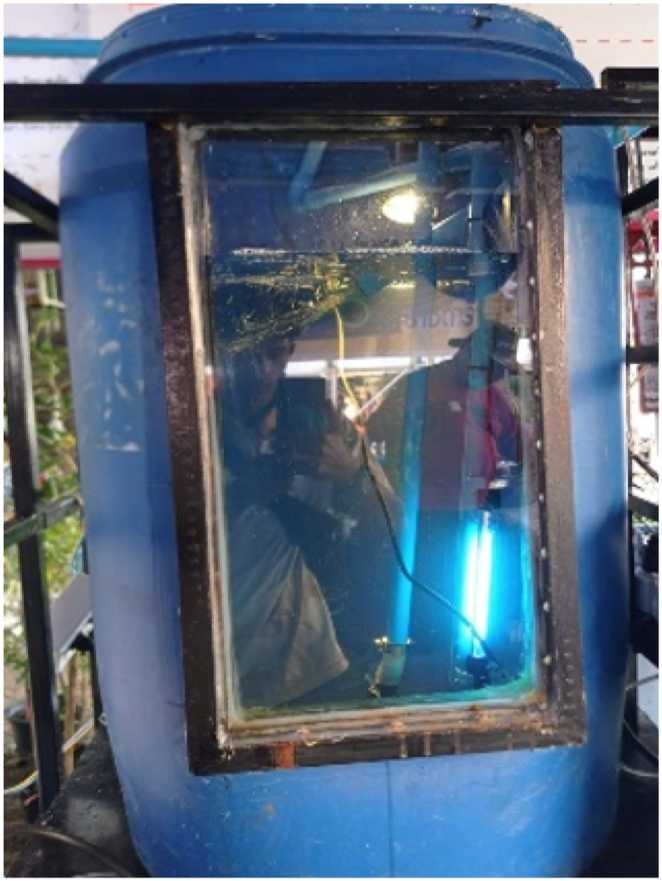
Water treatment system of tank 3 (disinfect and increase DO).

The water treatment system comprises three tanks, as depicted in Fig. 2, Fig. 3, Fig. 4. Tank 1 (Fig. 2(a–c)) utilizes three layers of filtration materials. The bottom layer, comprised of oyster shells renowned for their unique properties, plays a crucial role in water treatment by stabilizing pH, boosting alkalinity, and aiding in pollutant removal. This layer is commonly used for coarse filtration to support microbial presence. These microorganisms play a crucial role in treating water conditions due to their ability to break down organic matter and pollutants [18]. The middle layer contains lightweight pumice stones that can float on water. Using pumice stone for water filtration offers numerous benefits. Its porous structure facilitates microbial colonization and contaminant adsorption, ensuring thorough purification. It efficiently traps solids and organic matter, enhancing filtration and improving water quality. Additionally, its natural adsorption properties make it effective in removing pollutants. Pumice stone is cost-effective, renewable, and eco-friendly, making it ideal for sustainable water treatment [19]. The top layer comprises a fine filter mat that maintains the pH value of the water without causing any changes. The filter mat is positioned to prevent sludge leakage into the second treatment tank, which could result from pH fluctuations induced by sludge. Hence, the system must initially filter the sludge at the first tank as the initial step [20]. This layer provides a habitat for an ample number of microorganisms to efficiently remove contaminants. Being lightweight and easy to clean, the filter mat is favored as a filtration material. Tank 2 (Fig. 3(a and b)) encompasses two layers of filtration materials for finer waste filtration. The bottom layer employs 1–8 special filter mats to prevent pH value fluctuations in the water. A uniform filter mat, comprising glass and cellulose fibers bonded with polyurethane resin, was effectively manufactured through a straightforward wet-laying technique. Following roughening treatment, the mats exhibited an even distribution of pore sizes, averaging 5.05 μm. Additionally, the mats displayed discernible differences in wettability towards water and oil when exposed to air. Their oil wettability underwater was finely balanced between oleophilicity and oleophobicity, a critical aspect for their suitability in coalescence applications [21]. It is designed to provide a suitable habitat for microorganisms to remove waste matters from the pond. The top layer consists of 2–3 thick filter fibers that efficiently filter contaminants in the pond, resulting in clean and clear water without any sediments. There are filter panels made of rough fiber material designed to capture solid particles from the water in the aquaculture system. Various sediment waste residues were disposed of by installing valves at the bottom positions of tanks 1–3. The tank 3, known as the disinfection system (Fig. 4), facilitates the flow of water from the second tank through a UV (Ultraviolet) disinfection system. The system utilized UV-C type lamps with a wavelength range of 200–280 nm, possessing germicidal properties. The lamps were of the compact type with G23 bases and 9W in size. UV light lamp is commonly used in water treatment systems to reduce harmful bacteria and pathogens [22]. UV light emitted by the lamp damages the DNA and RNA of microorganisms such as bacteria, viruses, and protozoa. When these microorganisms are exposed to UV light, their ability to replicate and cause infections is greatly reduced or eliminated. When harmful bacteria are killed off, the organic matter they consume is no longer metabolized, leading to a decrease in biochemical oxygen demand (BOD) in the water. As a result, more dissolved oxygen (DO) becomes available for aquatic organisms such as fish and prawn. Subsequently, the water is returned to the pond after passing through these three tanks.

**Fig. 5:**
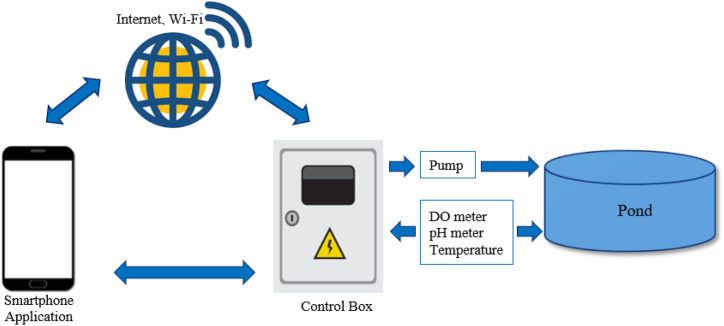
Quality control system of smart water management system.

The smart water quality control system incorporates sensors for DO, pH, and temperature, as well as a skimmer and an aeration pump in the pond. The operation of water management system begins when the oxygen dissolution sensor detects dissolved oxygen levels in the water below 8 mg per liter. It sends a signal to the microcontroller board to activate the air pump to aerate the water in the pond. When the dissolved oxygen level exceeds 9 mg per liter, the microcontroller board commands the pump to stop operating. Additionally, the pH meter sends a signal to the microcontroller board when the pH level falls below 7 or rises above 9, prompting the pump to draw water and sludge from the pond into the treatment system. These components are interconnected with a Raspberry Pi4 microcontroller board and an Internet of Things system, allowing for water quality monitoring through a smartphone, as depicted in Fig. 5. The control box of the smart water management system facilitates the integration and management of these components.

**Fig. 6:**
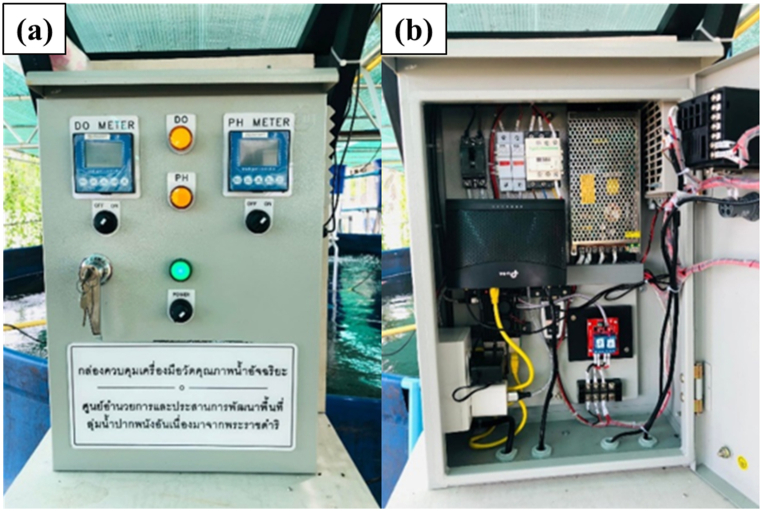
Control box of smart water management system. (a) Control box and (b) inside control box.

**Fig. 7:**
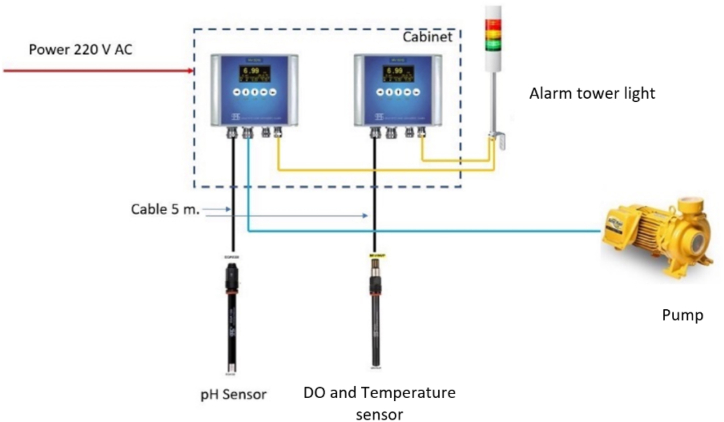
Water quality sensor system.

Fig. 6(a and b) depicts the control box of the smart water management system designed for giant freshwater prawn farming in Pak Phanang. The control box operates based on the following principle: when the sensor (Fig. 7) detects a dissolved oxygen (DO) level below 8 mg/L [23,24], the aeration pump activates automatically to increase oxygen levels until the sensor registers a new DO reading of 9 mg/L. At that point, the pump ceases operation. If the pH falls outside the optimal range of 7–9 [25], the system commands the pump to automatically transfer water and sediments into the filtration and treatment system to adjust the water condition to the appropriate pH.

**Fig. 8:**
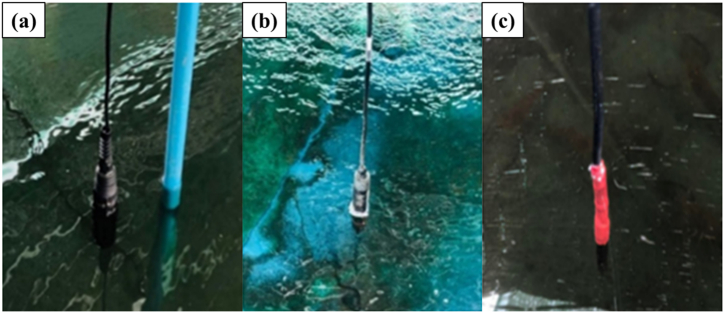
Using sensors to measure water quality. (a) pH sensor, (b) DO sensor and (c) temperature sensor.

**Fig. 9:**
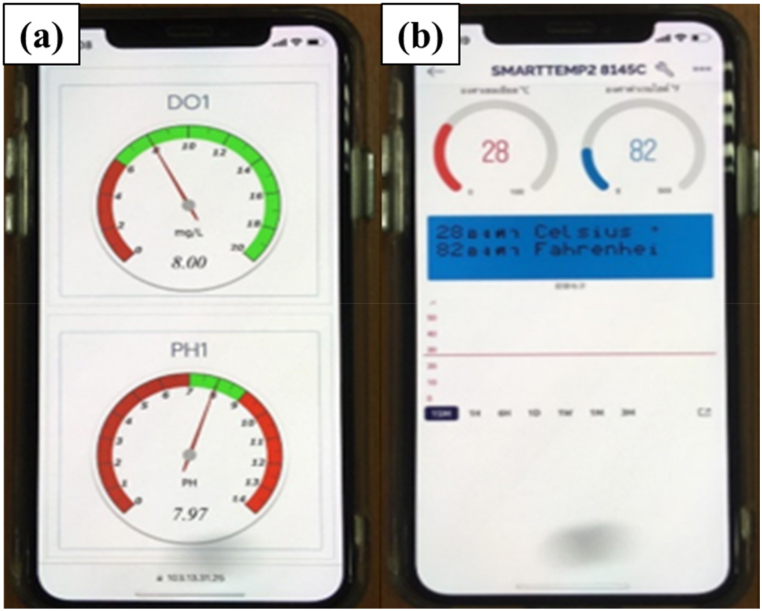
The real time monitor of automatic water management system by using IoT. (a) DO and pH and (b) temperature.

The temperature, DO, and pH sensors are utilized to measure the water parameters within the pond (Fig. 8(a–c)). These measurements are displayed in real-time numerical values on a smartphone application, as illustrated in Fig. 9(a and b). This enables continuous monitoring of the water quality in the river prawn pond, allowing for easy tracking of its status. In the event of any issues with the treatment system, immediate corrective actions can be taken. Moreover, all precise data are securely stored on servers, allowing for retrospective analysis of the culture outcomes and identification of factors impacting the growth of freshwater prawns in the pond. The water management system automatically adjusts water quality levels when it detects them falling below the designated threshold.

**Fig. 10:**
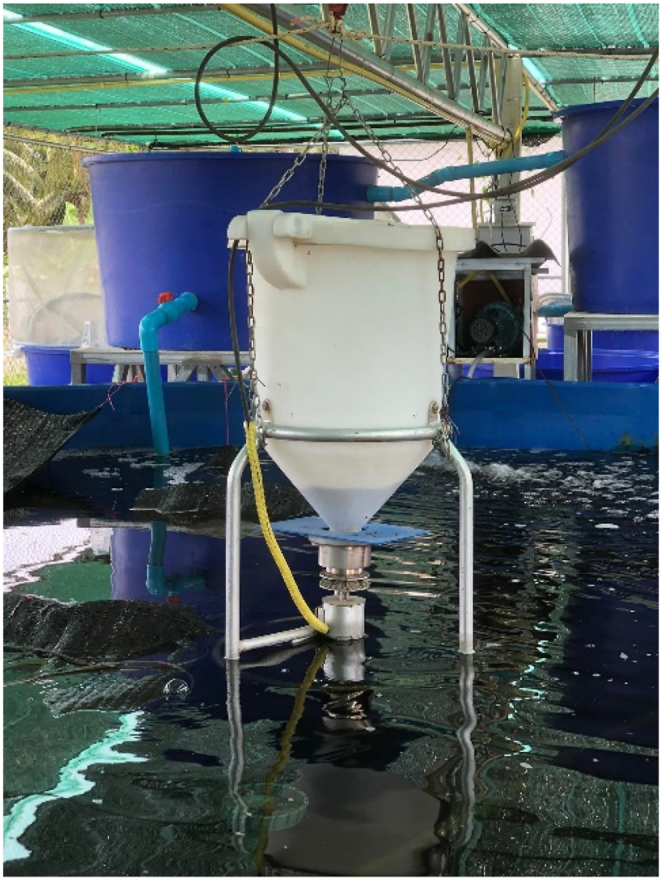
Automatic feeding system.

The automated feeding system (Fig. 10) incorporates a feeding hopper that can be programmed and timed to dispense the designated amount of food for the prawns during each feeding session, eliminating the need for manual feeding. The feeding schedule is set to occur three times a day at specified times: 7:00 a.m., 11:00 a.m. and 3:00 p.m.

### 2.2 Experiments for the collection of the data

This study utilized populations of giant freshwater prawns obtained from natural water sources in the Pak Phanang watershed, spanning a distance of 12,000 m from Sai Mak Sub-district, Chian Yai District to Hu Long Sub-district, Pak Phanang District, Nakhon Si Thammarat Province. The purposive sample consisted of approximately 270 Pak Phanang giant freshwater prawns, measuring 10–12 cm in length and weighing 24–30 g per individual. The prawns were reared in a prototype pond for a duration of 120 days.

### 2.3 Ethics statement

The handling of animals and the study protocol received approval from the Walailak University Institutional Animal Care and Use Committee (WU-IACUC) with the protocol number WU-ACUC-65058.

## 3 Results and discussion

**Fig. 11:**
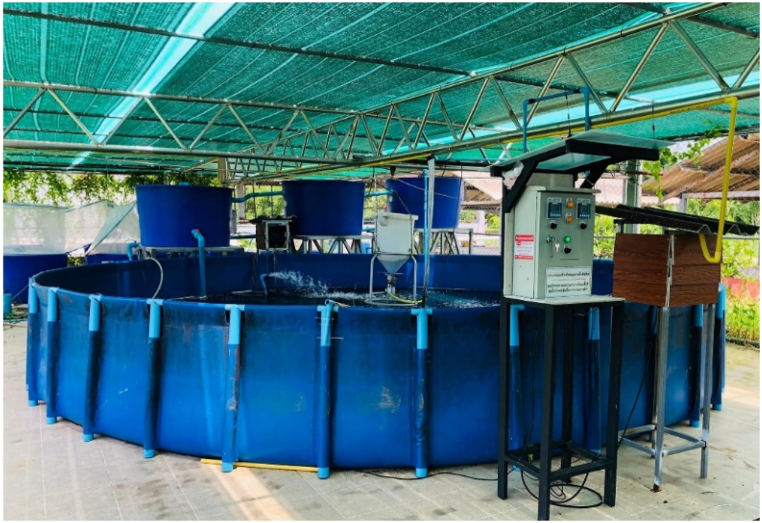
Prototype of smart water management system for farming Pak Phanang giant freshwater prawns.

We have developed a prototype of a smart water management system to regulate water quality, which directly impacts the growth of Pak Phanang giant freshwater prawns in a pond. The entire system consists of ponds, a water treatment system (comprising 3 tanks), and smart water quality control systems, as illustrated in Fig. 11. This research focuses on the prototyping of a smart water management system specifically designed for freshwater prawn farming. It serves as a valuable model for the application and advancement of water systems within pond environments. The system utilizes a chemical-free treatment approach to accurately determine the appropriate water quantity required for each production cycle. This closed system enables precise control over water quality. However, it should be noted that the quality of water intake from external sources cannot be controlled within the farming system. Notably, the remaining water from the prawn culture in the prototype system retains a high-quality state, free from any waste. This environmentally friendly characteristic is a significant advantage of the system.

**Fig. 12:**
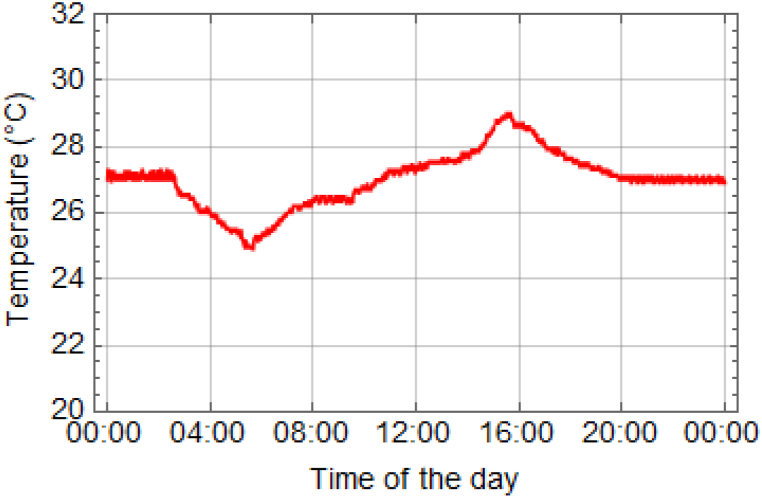
Temperature of water in 24 h.

**Fig. 13:**
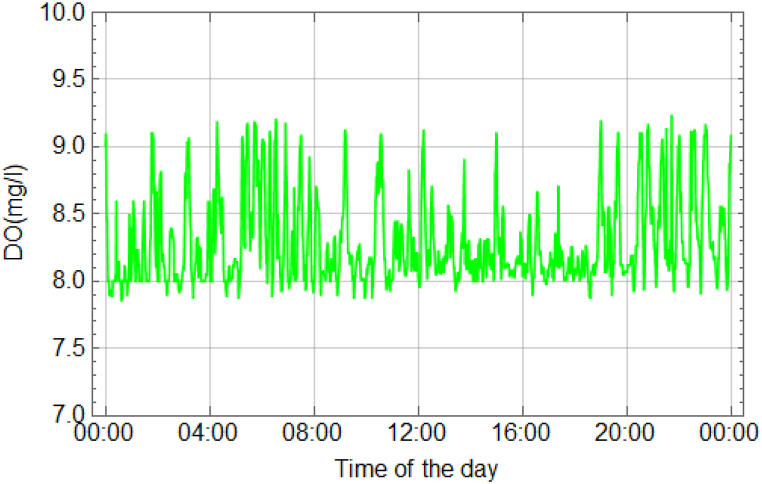
DO of water in 24 h.

**Fig. 14:**
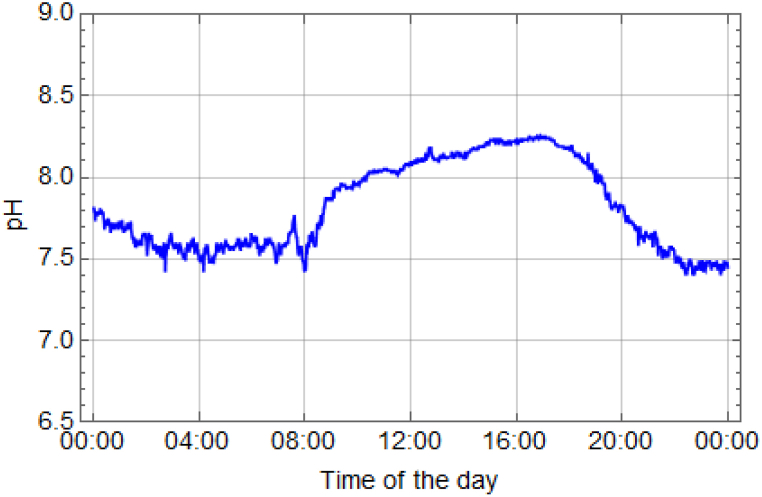
Changes in water pH during 24 h.

In the freshwater prawn pond, various parameters were assessed, including temperature, dissolved oxygen (DO), and pH values. The temperature, dissolved oxygen (DO) and pH levels of the water were monitored over the course of 120 days during the cultivation of giant freshwater prawns. (see Supporting Information Table S1). The data were collected over a 24-h period, with measurements recorded every minute. Detailed explanations of the experimental results for each parameter can be found in Fig. 12, Fig. 13, Fig. 14. The temperature measurements depicted in Fig. 12 revealed that a decrease in water temperature was observed during the nighttime period, with a minimum recorded at 25.0 °C. Subsequently, an increase was noted during the daytime period, reaching a maximum of 29.0 °C, with an average of 27.0 °C. These temperature variations were found to facilitate optimal prawn growth throughout the rearing process. It was previously indicated by research that the suitable temperature range for rearing river prawns is between 28 and 31 °C [6,23,25]. Additionally, rearing prawns at temperatures exceeding 35 °C was found to decrease prawn immunity and result in higher mortality rates [7]. The solubility of oxygen in water and pH were inversely proportional to water temperature, with an increase in temperature reducing the water's ability to dissolve oxygen. Higher water temperatures increased the likelihood of water dissociating into H+ ions, thereby lowering the pH level. It was indicated that the water temperature is suitable for prawn cultivation. The presence of a roofed house facilitated ventilation in all directions, contributing to the optimal conditions within the pond.

Fig. 13 illustrates the DO levels in the pond. The minimum DO record was 7.85 mg/L, while the maximum was 9.24 mg/L, with a mean of 8.29 mg/L. Adequate DO levels are crucial for prawn growth [26]. The automatic operation of the oxygen replenishment system in the prawn rearing pond was initiated when the DO sensor recorded a value lower than 8 mg/L. The system was configured at this level because it represents a high DO value; even if the DO concentration in the rearing pond decreases below this threshold, it would still not pose a danger to the river prawns. Various research studies have indicated that the suitable DO level for rearing river prawns should not fall below 5 mg/L. Therefore, the set DO value is considered safe, even if the system takes longer than necessary to replenish oxygen, as dissolved oxygen in water is one of the most influential environmental factors affecting various physiological processes of aquatic animals. A low DO concentration in water adversely affects the normal behavior and physiology of prawns, such as survival, respiration, circulation, feeding, metabolism, and growth [8]. A study in Bangladesh, involving 120 days of prawn rearing using an IoT system to monitor and control water quality similar to our research, found that the recorded DO averaged 6.90 mg/L, resulting in a survival rate of 80–90 % [24].

The results presented in Fig. 14 demonstrate a diurnal pattern in pH levels, with a peak value of 8.26 during the day and a minimum of 7.40 at night. The mean pH was measured at 7.82. This phenomenon occurred due to sunlight exposure during the daytime, stimulating photosynthesis in aquatic plants and phytoplankton, which utilize carbon dioxide for photosynthesis. Aquatic plants and phytoplankton help to reduce the concentration of carbon dioxide in the water [27]. Typically, in freshwater ponds, the pH level fluctuates from 6.6 to 10.2 due to the removal of carbon dioxide through photosynthesis by plants during the daytime and the release of carbon dioxide gas from the respiration of plants and animals during the nighttime [28]. Controlling the pH in prawn rearing ponds is crucial. In closed-system prawn rearing ponds, pH control is easier compared to natural earthen ponds. Studies conducted in various areas have shown that rainfall affects the pH level in prawn rearing ponds, particularly acidic rainfall [29], which leads to pH reduction in natural freshwater environments [30]. In prawn culture, it is essential to maintain the water pH within the optimal range of 7.0–8.5. Excessive or fluctuating pH levels during the day can induce stress in prawns, hindering their growth and compromising their immune system, rendering them more susceptible to infections [5,31]. To address this, a chemical-free treatment system was implemented to effectively manage the circulating water within the prawn ponds. This approach enables precise control over water usage for each production cycle and ensures efficient water quality management. Moreover, the freshwater prawn pond operates as a closed system, receiving no water input from external sources. Earlier studies have extensively investigated the variables critical for the survival of freshwater prawns, including dissolved oxygen (DO), water temperature, and pH levels. The findings of this research align with previous studies [4,6,23,25,32].

**Fig. 15:**
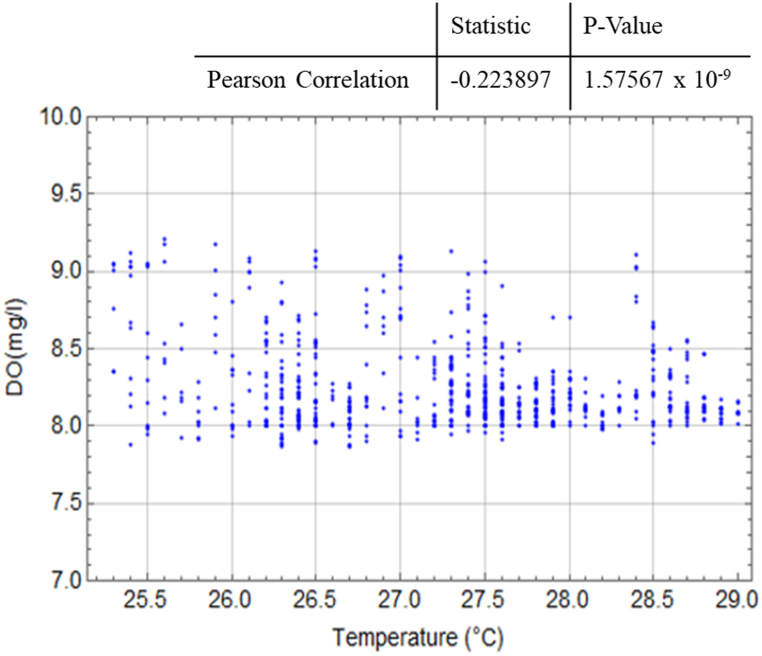
Relationship between temperature and DO in the prawn pond.

**Fig. 16:**
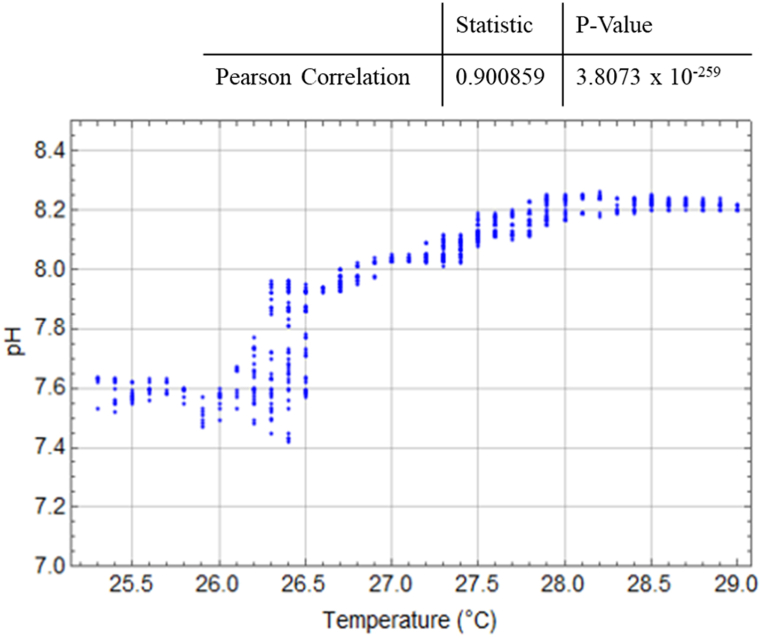
Relationship between temperature and pH in the prawn pond.

Fig. 15 displays the relationship between temperature and DO values, while Fig. 16 illustrates the relationship between temperature and pH values. To assess these correlations, Pearson's correlation statistics were conducted using Wolfram Mathematica [33]. The statistical analysis reveals that the temperature and DO values have a correlation coefficient of −0.224, indicating a low-level negative correlation that is highly statistically significant (p < 0.05). On the other hand, the temperature and pH values exhibit a correlation coefficient of 0.901, indicating the highest level of correlation with a consistent direction that is also highly statistically significant.

**Fig. 17:**
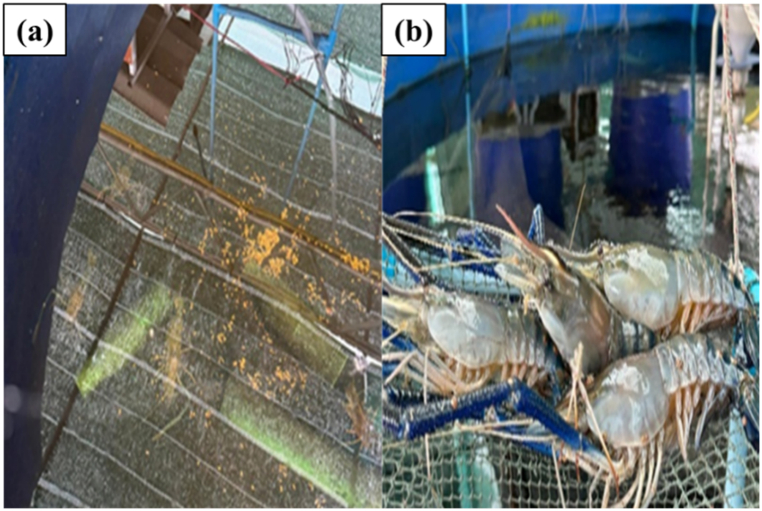
Cultivation of giant freshwater prawns in a prototype of a smart water management system. (a) Feeding behavior of giant freshwater prawns (b) the giant freshwater prawns ready to be harvested for sale.

Fig. 17(a and b) illustrates the cultivation of giant freshwater prawns using the prototype system of a smart water management system, which offers convenience for prawn farmers in their care-taking activities. This system allows farmers to allocate time for other tasks while providing them with the ability to monitor crucial measurements using a smartphone at any given time. Additionally, corrections and adjustments can be made through the smartphone interface, enhancing efficiency and ease of management [24,25,[34], [35], [36], [37], [38], [39], [40], [41]].

**Fig. 18:**
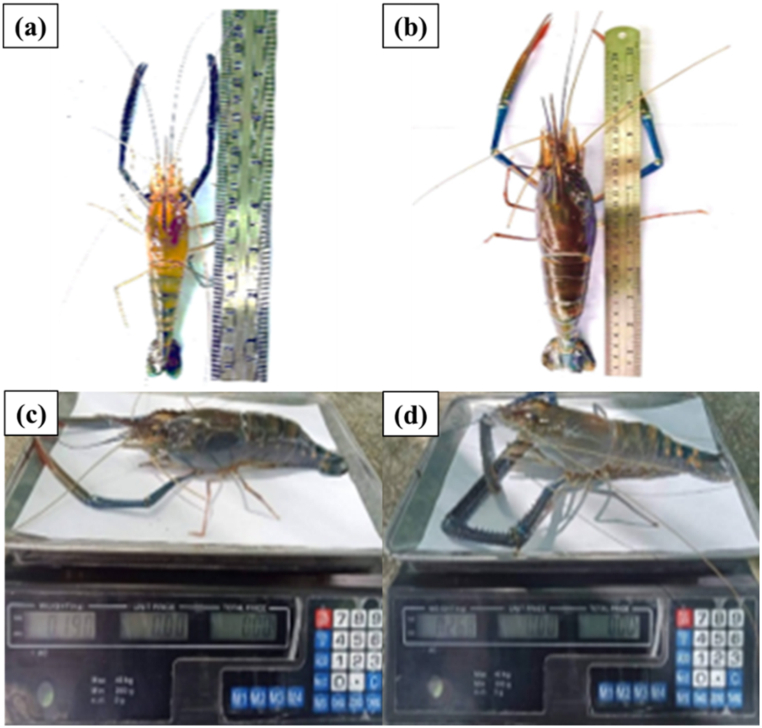
Giant Freshwater Prawn. (a) before, (b) after, (c) 190 g of prawn and (d) 268 g of prawn.

**Fig. 19:**
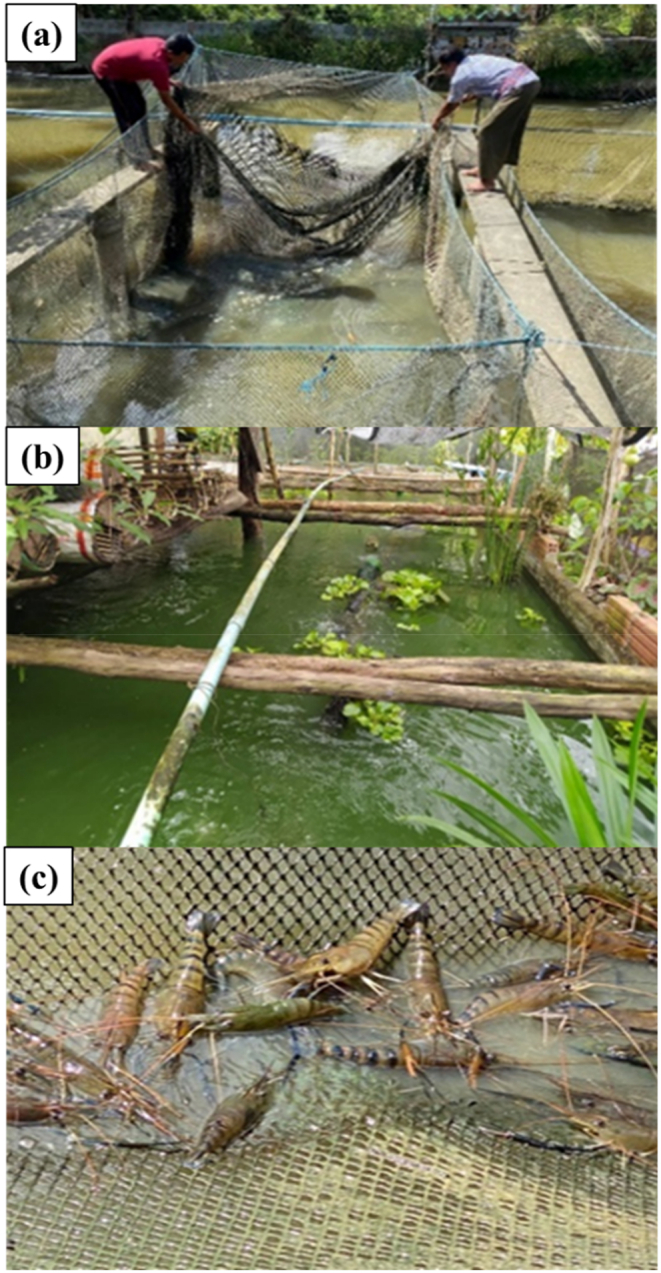
Cultivation of giant freshwater prawns by local fishermen in Pak Phanang District, Nakhon Si Thammarat Province. (a) Characteristics of giant freshwater prawns caught in ponds, (b) Giant freshwater prawn farms operated by local fishermen and (c) Giant freshwater prawns harvested by local fishermen.

According to Fig. 18(a–d), a total of 270 Pak Phanang giant freshwater prawns were stocked in the pond, with an average size of 10–12 cm in length and 24–30 g in weight per individual. After a rearing period of 120 days, 252 Pak Phanang giant freshwater prawns were successfully obtained, exhibiting a high survival rate of 93.3 %. These prawns reached an average length of 20–25 cm and a weight of 190–268 g per prawn, and they remained disease-free. Fig. 19(a–c) depicts the prawn farming practices employed by local fishermen, who encounter various challenges. Firstly, they struggle with maintaining water quality control. Secondly, it requires constant monitoring of water conditions and prawn health, necessitating periodic manual adjustment of aeration systems. Thirdly, there is a lack of adherence to hygiene standards in the production of food products. Additionally, local fishermen face several other problems. Lastly, the machines involved in prawn farming operations require prolonged and intense operation throughout a crop cycle of 3–4 months, thereby increasing production costs.

The system operates normally as programmed to automatically treat water when the system detects water quality below the specified threshold. Throughout the 120-day experimental period, resulting in the production of prawns with a high survival rate of 93.3 %, the system encountered occasional issues with internet signal and intermittent electrical disruptions. These disruptions were infrequent due to the location of the experiment site along the coast of the Gulf of Thailand, which may be affected by rain and storms (though not impacting river prawn farming). In such instances, system administrators may need to intervene promptly, with offline notifications provided to them via smartphone screens to alert them of internet or electrical issues, ensuring uninterrupted water treatment. Backup power generators may be utilized to promptly address such occurrences. Reviews of the water management system for prawn farming were consolidated or condensed into Table 1. Overall, non-clay ponds, as demonstrated in this work, offer significant advantages over clay ponds for freshwater giant prawn farming, including improved cleanliness, cost-effectiveness, contamination control, bacteria management, and disease prevention, making them a preferred choice for many farming operations aiming to optimize productivity and sustainability. Cleanliness: Non-clay ponds typically have smoother surfaces, making them easier to clean and reducing the accumulation of organic matter and waste. The smooth surfaces of non-clay ponds minimize areas where bacteria and pathogens can proliferate, resulting in cleaner water conditions compared to clay ponds. Cost: While non-clay ponds may have higher initial construction costs compared to clay ponds, they often require less maintenance and repair over time. The long-term cost savings associated with reduced maintenance and improved efficiency can outweigh the initial investment for non-clay ponds. Contamination Control: Non-clay ponds, especially those with liners or sealed surfaces, provide better control over contamination by preventing pollutants, chemicals, and pathogens from leaching into the environment. The use of liners in non-clay ponds creates a barrier that maintains water quality and ecosystem health, reducing the risk of contamination compared to clay ponds. Bacteria Management: The smooth, impermeable surfaces of non-clay ponds minimize habitat for bacteria and pathogens, reducing the risk of disease outbreaks among prawn populations. Improved water quality management in non-clay ponds helps control bacterial growth and maintains optimal conditions for shrimp health and growth. Disease Prevention: Non-clay ponds with enhanced water quality management and reduced exposure to external contaminants help prevent the spread of diseases among shrimp populations. Enhanced biosecurity measures, such as pond liners and controlled access, further reduce the risk of introducing pathogens and diseases to prawn farms in non-clay ponds compared to clay ponds.

**Table 1:** The reviews of water management system for prawn farming.

Water management system	Reference
The temperature, pH, and dissolved oxygen parameters were measured and monitored for 24 h. Continuous monitoring was carried out by the inspection system if sensor values fell below the predefined threshold. Notification messages along with status updates were sent to the mobile device by the system.	[10]
In the shrimp pond, measuring 180 cm in height, 180 cm in length, and 60 cm in width, an automatic sensor system was installed to monitor the water temperature and an ultrasonic sensor was employed to assess the water level. An automated water treatment system was instructed to control water circulation when water quality deviated from predefined values.	[36]
The experiment was conducted in a rectangular concrete tank for a duration of 120 days, with the water quality parameters including dissolved oxygen, pH, and temperature being monitored and controlled.	[42]
A cylindrical tank made of polyethylene fabric with a thickness of 0.7 mm and a diameter of 6 m, and a depth of 70 cm was used. Factors affecting the growth of freshwater prawns in the prototype pond were controlled by implementing an intelligent water management system. This was achieved through an IoT system capable of monitoring the pond water quality by continuously observing temperature, pH, and dissolved oxygen (DO) levels. When the DO sensor detected levels below 8 mg per liter, the oxygen replenishment pump automatically operated to increase oxygen levels until the sensor registered the desired DO level again.	This work

## 4 Conclusions

The prototype of a smart water management system designed for Pak Phanang giant freshwater prawn farming incorporates key components such as river prawn ponds, a water treatment system, an automatic feeding system, an automated water quality control system with sensors for monitoring parameters such as DO, pH, and temperature, as well as an IoT system that enables real-time monitoring of measurement results via smartphone. This comprehensive system significantly reduces the time and effort required by farmers for continuous cultivation of giant freshwater prawns. The research findings indicate a high survival rate and the production of quality large prawns at optimal market size using this system. It serves as an innovative solution for water management, enabling control over water conditions and maintaining specified water quality standards at all times. Although the initial cost of implementation may be substantial, there are no additional maintenance costs once the system is installed. Thus, this prototype offers a viable and effective solution for giant freshwater prawn farmers. It can be further developed by adapting the system for pond-raised prawn farming and monitoring water quality in earthen ponds. With the intelligent water management system, electricity consumption can be reduced by transitioning to alternative energy sources such as solar power and wind, thereby decreasing expenses, labor costs, preserving the environment, and improving resource utilization from the treatment system to zero waste.

## Data availability statement

Data included in article/supp. Material/referenced in article. No additional information is available for this paper.

## CRediT authorship contribution statement

**Panyapong Songpayome:** Writing – original draft, Software, Methodology, Conceptualization. **Supapron Sutin:** Supervision, Conceptualization, Methodology. **Warawut Sukmak:** Supervision, Conceptualization, Methodology. **Uraiwun Wanthong:** Supervision, Conceptualization, Methodology. **Nunticha Limchoowong:** Supervision, Methodology, Conceptualization. **Phitchan Sricharoen:** Supervision, Methodology, Conceptualization. **Panjit Musik:** Writing – review & editing, Supervision, Software, Conceptualization, Data curation, Project administration.

## Declaration of competing interest

The authors declare that they have no known competing financial interests or personal relationships that could have appeared to influence the work reported in this paper.
