# Supplementary material for: A prototype pond water management system (dissolved oxygen, pH and temperature) for giant freshwater prawn farming in Pak Phanang, Southern Thailand
Source: Heliyon. 2024 May 14;10(10):e31231. doi: 10.1016/j.heliyon.2024.e31231 (PMC11129002; doi:10.1016/j.heliyon.2024.e31231)
Supplement: Multimedia component 1 [file mmc1.docx]

**Supporting Information**

**A prototype pond water management system (dissolved oxygen, pH and temperature) for giant freshwater prawn farming in Pak Phanang, Southern Thailand**

**Table S1** The temperature, dissolved oxygen (DO), and pH levels of the water were monitored over the course of 120 days during the cultivation of giant freshwater prawns. (Submit a daily report at 10:00 AM.)

| **DAY** | **Temp. (°C)** | **DO (mg/L)** | **pH** |
| --- | --- | --- | --- |
| 1 | 26.50 | 8.02 | 9.13 |
| 2 | 26.50 | 8.05 | 8.93 |
| 3 | 26.00 | 8.19 | 8.80 |
| 4 | 26.00 | 8.27 | 8.71 |
| 5 | 25.50 | 8.26 | 7.67 |
| 6 | 25.00 | 8.26 | 8.38 |
| 7 | 25.00 | 8.09 | 8.72 |
| 8 | 25.50 | 8.05 | 8.65 |
| 9 | 26.00 | 8.08 | 8.56 |
| 10 | 26.00 | 8.15 | 8.49 |
| 11 | 26.00 | 8.15 | 8.56 |
| 12 | 26.00 | 8.19 | 8.50 |
| 13 | 26.00 | 8.23 | 8.52 |
| 14 | 26.50 | 8.21 | 8.47 |
| 15 | 26.50 | 8.14 | 8.68 |
| 16 | 27.00 | 8.14 | 8.53 |
| 17 | 27.00 | 8.09 | 8.47 |
| 18 | 27.00 | 8.01 | 8.46 |
| 19 | 27.00 | 8.01 | 8.39 |
| 20 | 27.00 | 8.06 | 8.30 |
| 21 | 27.00 | 8.00 | 8.50 |
| 22 | 27.50 | 8.99 | 7.56 |
| 23 | 27.80 | 8.75 | 8.13 |
| 24 | 28.00 | 8.06 | 8.22 |
| 25 | 28.50 | 8.26 | 8.24 |
| 26 | 29.00 | 8.65 | 8.18 |
| 27 | 29.00 | 8.01 | 8.17 |
| 28 | 28.50 | 8.05 | 8.18 |
| 29 | 28.50 | 8.11 | 8.41 |
| 30 | 28.00 | 8.19 | 8.13 |
| 31 | 27.00 | 8.17 | 8.53 |
| **DAY** | **Temp. (°C)** | **DO (mg/L)** | **pH** |
| 32 | 27.10 | 8.05 | 8.52 |
| 33 | 27.20 | 8.00 | 8.23 |
| 34 | 27.10 | 8.13 | 8.46 |
| 35 | 26.90 | 8.00 | 7.79 |
| 36 | 27.10 | 8.00 | 8.52 |
| 37 | 27.00 | 8.18 | 8.23 |
| 38 | 26.80 | 8.04 | 8.33 |
| 39 | 26.80 | 8.17 | 8.42 |
| 40 | 26.80 | 8.02 | 8.19 |
| 41 | 26.70 | 8.04 | 8.38 |
| 42 | 26.80 | 8.00 | 8.25 |
| 43 | 26.70 | 8.01 | 8.17 |
| 44 | 26.70 | 8.02 | 8.37 |
| 45 | 26.50 | 8.00 | 8.12 |
| 46 | 26.60 | 8.05 | 8.49 |
| 47 | 28.00 | 8.01 | 8.56 |
| 48 | 27.50 | 8.09 | 8.37 |
| 49 | 27.50 | 8.00 | 8.76 |
| 50 | 27.50 | 8.01 | 8.10 |
| 51 | 27.00 | 8.22 | 7.96 |
| 52 | 27.00 | 8.08 | 8.13 |
| 53 | 27.00 | 8.08 | 7.34 |
| 54 | 27.00 | 8.03 | 8.31 |
| 55 | 27.00 | 8.07 | 8.45 |
| 56 | 27.00 | 8.09 | 8.30 |
| 57 | 27.00 | 8.20 | 8.45 |
| 58 | 27.00 | 8.17 | 8.22 |
| 59 | 26.50 | 8.03 | 8.16 |
| 60 | 26.70 | 8.00 | 8.27 |
| 61 | 26.60 | 8.13 | 8.27 |
| 62 | 26.60 | 8.26 | 8.38 |
| 63 | 26.60 | 8.00 | 8.23 |
| 64 | 26.50 | 8.07 | 10.73 |
| 65 | 26.50 | 8.27 | 9.73 |
| 66 | 26.60 | 8.00 | 8.17 |
| 67 | 26.50 | 8.13 | 8.31 |
| 68 | 26.60 | 8.00 | 8.19 |
| 69 | 26.50 | 8.04 | 8.24 |
| 70 | 26.50 | 8.03 | 8.22 |
| 71 | 26.50 | 8.00 | 8.40 |
| **DAY** | **Temp. (°C)** | **DO (mg/L)** | **pH** |
| 72 | 26.60 | 8.08 | 7.97 |
| 73 | 27.00 | 8.00 | 8.17 |
| 74 | 27.50 | 8.00 | 8.26 |
| 75 | 27.80 | 8.15 | 8.27 |
| 76 | 28.00 | 8.00 | 8.04 |
| 77 | 28.50 | 8.06 | 8.26 |
| 78 | 29.00 | 8.00 | 8.26 |
| 79 | 29.00 | 8.00 | 8.24 |
| 80 | 28.50 | 8.06 | 8.38 |
| 81 | 28.50 | 8.00 | 8.27 |
| 82 | 28.00 | 8.03 | 8.45 |
| 83 | 27.00 | 8.11 | 8.49 |
| 84 | 26.80 | 8.10 | 8.46 |
| 85 | 26.80 | 8.15 | 8.55 |
| 86 | 26.80 | 8.02 | 8.71 |
| 87 | 26.70 | 8.15 | 8.16 |
| 88 | 26.80 | 8.18 | 7.40 |
| 89 | 26.70 | 8.03 | 7.20 |
| 90 | 26.70 | 8.10 | 7.37 |
| 91 | 26.50 | 8.00 | 7.38 |
| 92 | 26.60 | 8.11 | 7.09 |
| 93 | 27.00 | 8.00 | 7.43 |
| 94 | 26.50 | 8.16 | 7.21 |
| 95 | 26.50 | 8.12 | 7.56 |
| 96 | 26.00 | 8.32 | 7.66 |
| 97 | 26.00 | 8.07 | 7.81 |
| 98 | 25.50 | 8.16 | 7.77 |
| 99 | 25.00 | 8.00 | 7.74 |
| 100 | 25.00 | 8.07 | 7.59 |
| 101 | 25.50 | 8.05 | 7.58 |
| 102 | 26.00 | 8.23 | 7.62 |
| 103 | 26.00 | 8.04 | 7.63 |
| 104 | 26.00 | 8.34 | 7.52 |
| 105 | 26.00 | 9.01 | 7.96 |
| 106 | 26.00 | 8.00 | 7.97 |
| 107 | 26.50 | 8.24 | 7.98 |
| 108 | 26.50 | 8.48 | 8.00 |
| 109 | 27.00 | 8.84 | 7.98 |
| 110 | 27.50 | 8.88 | 7.98 |
| 111 | 27.80 | 8.88 | 8.01 |
| **DAY** | **Temp. (°C)** | **DO (mg/L)** | **pH** |
| 112 | 28.00 | 8.63 | 8.01 |
| 113 | 28.50 | 8.39 | 8.15 |
| 114 | 29.00 | 8.04 | 8.16 |
| 115 | 29.00 | 8.00 | 8.15 |
| 116 | 28.50 | 8.00 | 8.16 |
| 117 | 28.50 | 8.00 | 8.19 |
| 118 | 28.00 | 8.00 | 8.20 |
| 119 | 28.00 | 8.00 | 8.19 |
| 120 | 29.00 | 8.00 | 8.22 |
